# Multiple-biomarker approach in the assessment of bisphenol A effect on the grooved carpet clam *Ruditapes decussatus* (Linnaeus, 1758)

**DOI:** 10.1186/s40850-024-00211-1

**Published:** 2024-08-12

**Authors:** Ola Mohamed Nour, Salwa A. El-Saidy, Aml Z. Ghoneim

**Affiliations:** 1 Department of Biology and Geology, Faculty of Education, Alexandria University, Alexandria, 21526 Egypt; 2 Zoology Department, Faculty of Science, Damanhour University, Damanhour, Egypt

**Keywords:** Bisphenol A, *Ruditapes decussatus*, Oxidative stress, Lipid peroxidation, Antioxidant biomarkers, Acetylcholinesterase, DNA damage, Digestive gland histology

## Abstract

**Background:**

Bisphenol A (BPA), a plastic additive monomer, is among the most highly produced chemicals worldwide, and is broadly used in many industries, such as food and beverage containers, milk bottles, and paper products. Previous studies demonstrated that BPA has potential toxicity to aquatic organisms, causing endocrine disturbance and behavioural disorders. The current work aimed to determine the toxic impacts of BPA on the edible marine clam *Ruditapes decussatus* considering a multi-biomarker approach (mortality, biochemical studies, DNA strand breaks using comet assay, and histopathological examinations with semi-quantitative and quantitative histopathological analyses). The clams were exposed under laboratory conditions to three concentrations of BPA (0 “control”, 1, and 5 µg/L) for a period of 21 days. After the exposure period, BPA impacts were assessed in the digestive gland as a versatile and environmentally relevant organ for ecotoxicological studies.

**Results:**

In BPA-treated clams, mortality (10%) occurred only at the highest BPA concentration (5 µg/L). Biochemical impairments were detected in a concentration-dependent manner as a consequence of BPA exposure. There were significant increases in malondialdehyde (MDA) and glutathione (GSH) levels, while catalase (CAT) activity was significantly reduced. Our results revealed that BPA induced neurotoxicity in *R. decussatus*, as evidenced by the inhibition of acetylcholinesterase (AChE) activity in a dose-dependent manner. Furthermore, DNA damage was strongly induced as BPA levels increased. Additionally, our results have been affirmed by alterations in digestive gland tissues at BPA treatments, which consequently can impair the clam’s ability for food absorption; these alterations included mainly atrophic and necrotic digestive tubules, epithelial cell vacuolization, hemocyte infiltration, and intertubular fibrosis. Based on the data obtained from the semi-quantitative and quantitative histopathological analyses, the exposure of the clam’s digestive gland to BPA with concentrations of 1 and 5 µg/L for 21 days showed significant histopathological alterations compared with the control clams.

**Conclusion:**

The multi-biomarker approach used in the current study proved to be a useful tool for assessing the impact of diphenylmethane compounds, such as BPA. Water-borne BPA causes oxidative stress, neurotoxicity, genotoxicity, and deleterious effects on the clam digestive gland; all of these could deteriorate clam performance and health, causing tissue dysfunction.

## Background

The grooved carpet shell clam *Ruditapes decussatus* (Linnaeus, 1758) (Bivalvia: Veneridae) is a commercially valuable bivalve species inhabiting the Mediterranean Sea with high economic and ecological values [1, 2]. *R. decussatus*, like all bivalve species, is a sessile filter-feeder, making its tissues in direct contact with many chemical pollutants in the marine environment [3–5]. Due to its high capacity to tolerate these chemical pollutants, it can be used in ecotoxicological studies as a sensitive indicator species for pollution assessment [6].

Among the pollutants impacting the marine environment, bisphenol A (BPA; 2,2-bis-(4-hydroxyphenyl) propane) has achieved great interest. BPA is one of the world’s most widely used industrial pollutants [7, 8]. It is extensively used as a synthetic monomer in the production of polymeric materials, primarily polycarbonates and epoxy resins, that are introduced in plastics production, children’s toys, as well as used in food and beverage containers, like plastic bottles and baby feeding bottles [9–11]. Additionally, BPA is a dental sealant and thermal paper component and can be utilized as a stabilizer and antioxidant in manufacturing polyvinyl chloride plastics [12].

BPA poses detrimental biological impacts on marine organisms. It is one of aquatic systems’ most dominant phenolic pollutants [9, 13]. BPA is widely found in the marine ecosystem due to the continuous discharge of municipal and industrial wastes [14]. Although this compound slowly degrades in the environment, it can be leached from plastic products through photodegradation and biodegradation, and consequently, it can easily bioaccumulate in marine organisms, causing toxicity to these organisms [15–17]. Chronic exposure to BPA affects aquatic organisms, causing endocrine disruption, physiological alterations, behavioural and histological changes, cytotoxicity, immunotoxicity, neurotoxicity, genotoxicity, as well as reproductive toxicity [16, 18–22].

Ecotoxicological studies on bivalves use some potential tools to evaluate bivalve toxicity. These tools include estimation of oxidative damage, neurotoxic enzyme activity, and deoxyribonucleic acid (DNA) damage [23–26], in addition to examination of histopathological abnormalities [27].

The oxidative stress responses induced by chemicals were used as potential biomarkers to evaluate the effect of xenobiotics on aquatic animals [23]. The resulting oxidative damage can be assessed through the estimation of lipid peroxidation levels and enzymatic and non-enzymatic antioxidants in aquatic organisms [28]. However, the activities of antioxidant enzymes, like many other biochemical systems, are also influenced by various endogenous and exogenous factors [29], such as organism developmental stage and age [30], diet [31], reproductive cycle [32], temperature [33], salinity [34, 35], pH [35], and hypoxia/hyperoxia [36].

Reduced glutathione (GSH) and catalase (CAT) have been extensively used as defense antioxidant biomarkers and are involved in tissue redox balance [20, 37]. GSH is a non-enzymatic antioxidant involved in the second phase of metabolism and acts as an oxygen radical scavenger and an inhibitor of lipid peroxidation [38, 39]. It also acts as an important coenzyme for glutathione-S-transferase (GST) and glutathione peroxidase (GPx) [40, 41]. CAT is an enzyme antioxidant that widely serves as a sensitive oxidative stress biomarker [42]. CAT usually works in synergy with another antioxidant enzyme, superoxide dismutase (SOD) [43]. SOD protects cells by turning superoxide anion radicals into hydrogen peroxide (H_2_O_2_), which is further disintegrated into water and oxygen through CAT. Thus, these antioxidant enzymes constitute the first line of defense against free radicals in cells to detoxify ROS [43, 44]. Acetylcholinesterase (AChE) activity is a reliable indicator for assessing the neurotoxicity of environmental pollutants on aquatic bivalves [13, 26, 28]. This enzyme performs a vital function in cholinergic neurotransmission and is involved in maintaining the integrity and permeability of cell membranes during synaptic transmission and conduction [45]. It participates in breaking down acetylcholine (ACh) into choline and acetic acid at cholinergic synapses and neuromuscular junctions [26].

The identification of structural damage to DNA molecules within the bivalve genome serves as a significant biomarker for assessing the genotoxic consequences caused by environmental pollution. Destruction of the genome of the somatic cells can lead to progressive malfunction of tissues and organs, ultimately resulting in the death of the organism [46, 47]. The comet test is a commonly employed method for evaluating the genotoxicity of different pollutants in bivalves through the detection of DNA damage [24, 25].

Histopathology is an effective tool for evaluating the biological condition of coastal ecosystems through offering insights into the overall health of bivalves by examining their tissues [48–50]. The digestive gland is considered a primary site of homeostasis o﻿f the internal medium and is responsible for the bioaccumulation and detoxification of xenobiotics ingested with food particles. Also, it performs a vital role in intracellular digestion, hormone synthesis, and immunological defense [51, 52]. The digestive gland is rich in oxidizable substrates, such as fatty acids, which render this organ vulnerable to oxidative damage caused by free radicals [53]. Furthermore, it is widely recognized that the antioxidant defense system of the digestive gland aids in the elimination of excessive ROS caused by environmental pollutants [54]. The digestive gland is regarded as a primary organ of interest in toxicological studies due to the vulnerability of its epithelial cells to the deleterious effects of several contaminants in the marine environment [55].

Even though previous research has expressed worries about the adverse impacts of BPA on marine bivalve species [16, 56–59], there is a lack of available studies of BPA impact on *R. decussatus* clam. So, the current work was designed to explore the chronic toxic impact of BPA on *R. decussatus* clam through its survival, redox state, and detection of DNA damage in its digestive gland tissues. Moreover, it was conducted to identify the histopathological abnormalities in digestive gland tissues induced by chronic exposure to BPA.

## Materials and methods

### Collection and acclimation of the clam ***Ruditapes decussatus***

**Fig. 1:**
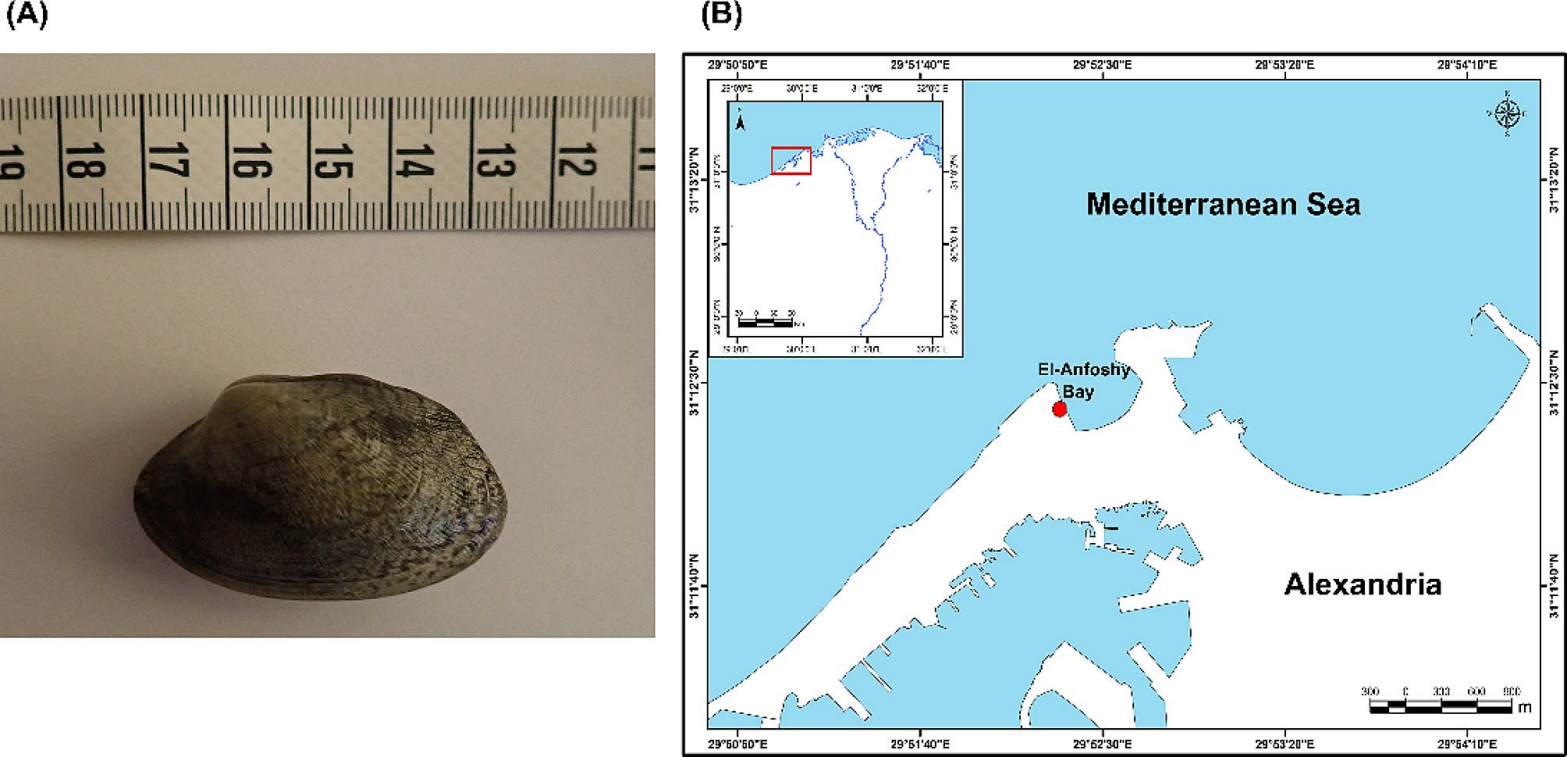
**(A)** The study organism, the grooved carpet clam *Ruditapes decussatus* (scale = 1 cm; the length between two successive long lines) and **(B)** Map showing the sampling site, El-Anfoshy Bay (red circle) in Alexandria city, Egypt, from which *Ruditapes decussatus* individuals were collected during October 2023. Small map (upper left corner) showing lower Egypt and the sampling site (red square)

About 200 individuals of the clam *R. decussatus* were collected in October 2023 in El-Anfoshy Bay (31° 12’ 24” N, 29° 52’ 18” E), Alexandria, Egypt **(**Fig. 1A**&B)**. The selection of the collecting site as a reference area was based on prior ecotoxicological studies conducted on marine bivalves [16, 60, 61]. The collected clams were placed in plastic boxes in well-aerated seawater obtained at the collection site and immediately transported to the laboratory at the Faculty of Education, Alexandria University. Upon arrival at the laboratory, clams were separated into three 20-litre glass aquaria (31 × 21 × 36 cm) covered with ~ 1 cm of clean sieved sand. The clams were acclimated for a period of 7 days in constantly aerated natural seawater renewed daily and kept at room temperature of 32 ± 1.5 ^°^C, salinity of 34 ± 0.5 psu, and photoperiod of 12 h:12 h (light: dark) before the beginning of the experiment. Over the acclimation period, clams were fed with fresh microalgae *Nannochloropsis oculata* (20,000 cells/ml) every two days.

### Experimental design

Only healthy clams, with intact well-closed shells and uniform size, were used in this assay. One hundred twenty clams measured 34.11 ± 0.22 mm shell length, 44.96 ± 0.21 mm shell width, and 22.11 ± 0.26 g weight, were randomly distributed into three groups with 40 individuals per group. In total, 15 five-litre glass aquaria were used as experimental units, with five replicates per group and eight clams per aquarium. Each aquarium (25 × 15 × 16 cm) was filled with 3 L of naturally continuously aerated seawater. The desired concentrations of BPA (CAS-RN: 80-05-7, C_15_H_16_O_2_, MW: 228.29 g/mol, 99% purity, Sigma-Aldrich, USA) [0 (control), 1, and 5 µg/L] were freshly prepared daily by dissolving BPA in the filtered seawater. Clams were exposed to BPA concentrations for a period of 21 days. The BPA concentrations and exposure period used in this work were chosen based on prior research on aquatic bivalves [16, 61, 62]. These concentrations are environmentally relevant concentrations based on the studies of Hatef et al. [63] and Ozhan and Kocaman [64]. In order to maintain BPA concentrations and to ensure water quality, seawater in every experimental unit was fully exchanged daily, and the concentrations of BPA were re-established. Phytoplankton (~ 1.57 × 10^6^ cells/L [65]) in the natural seawater, not removed by filtration, were the only food source for clams, and no additional food was added to clams during the exposure period [66–68].

### Survival status of the clam *Ruditapes decussatus*

Clams’ live or dead status was assessed daily during the experimental period. All clam individuals were gently checked. The presence of a gap between the two valves and no response to touch were considered as signs of death.

### Biochemical analysis in the clam ***Ruditapes decussatus*** digestive gland

At the end of the 21-day experimental period, clams (*n* = 3 individuals/concentration), i.e., one clam was chosen randomly per aquarium (*n* = 3 true replicates), were dissected using a scalpel to separate the digestive gland from the soft tissues. The digestive gland samples were homogenized three times with buffer solution (20 mM Tris buffer, 0.5 M sucrose, 0.15 M NaCl, pH 7.6) (1:9 *w/v*) in ice water for 30 s, using an electric mixer at 6,000 rpm. The homogenate was centrifuged at 9,000 ×g for 30 min at 4 ^°^C [69]. The supernatant was used for spectrophotometric evaluation of biochemical parameters utilizing a test kit obtained from Bio-Diagnostic Co. Dokki, Giza, Egypt. The protein content in samples was estimated following the Lowry et al. [70] method using bovine serum albumin (BSA, Sigma) as the standard. This analysis relied on reducing phosphomolybdic-phosphotungstic acid in the folin reagent under alkaline conditions. The absorbance of the supernatant was detected using a spectrophotometer (Evolution 300, Thermo Scientific, USA), at a wavelength of 540 nm.

### Determination of lipid peroxidation level

Lipid peroxidation was detected through the estimation of the malondialdehyde (MDA, CAT. NO. MD 25 29) level in the supernatant using the colorimetric method of Draper and Hadley [71]. This method relies on the MDA reaction with 2-thiobarbituric acid (TBA) in an acidic medium for 30 min at 95 ^°^C to form a TBA reactive product. The absorbance of the resulting pink-colored product can be determined at 534 nm. The MDA levels were presented as nmol/mg protein.

### Determination of reduced glutathione (GSH) level

The level of reduced glutathione (GSH, CAT. No. GR 25 11) was determined using a method that relies on the reduction of 5,5′-dithio-bis-2-nitrobenzoic acid (DTNB) by a sulfhydryl (-SH) group of glutathione, resulting in a yellow color. The reduced chromogen is directly proportional to the GSH level [72, 73]. The absorbance was recorded at 405 nm, and the GSH level was presented as µg/mg protein.

### Determination of catalase (CAT) activity

Catalase (CAT, CAT. No. CA 25 17) activity was estimated following the colorimetric method of Aebi [74]. CAT reacted with a predetermined amount of H_2_O_2_, and the reaction was halted precisely after one minute using sodium azide (NaN_3_) as a CAT inhibitor [75, 76]. In the presence of horseradish peroxidase (HRP), the remaining H_2_O_2_ reacted with 3,5-Dichloro-2-hydroxybenzene sulfonic acid (DHBS) and 4-aminophenazone (AP) to form a chromophore with a color intensity inversely proportional to the amount of CAT in the original sample. The absorbance was conducted at a wavelength of 520 nm, and its activity was presented as U/mg protein, where one unit of CAT is the amount of CAT in the sample that catalyses the dismutation of 1 µmol of hydrogen peroxide per minute.

### Determination of acetylcholinesterase (AChE) activity

The acetylcholinesterase (AChE, EC 3.1.1.7) activity was determined in the digestive gland homogenate supernatant following the Ellman et al. [77] method. AChE reacted with acetylcholine thioiodide (ACTI) as the first substrate and 5,5′-Dithio-bis-(2-nitrobenzoic acid) (DTNB) as a second substrate, giving a yellow-colored product. Absorbance was determined by spectrophotometer at 405 nm every two minutes. The AChE activity in the sample was presented as nmol of ACTI hydrolyzed/min/mg protein.

### Assessment of DNA damage in the digestive gland of *Ruditapes decussatus* clam using comet analysis

The genotoxicity of cells obtained from the digestive glands of *R. decussatus* (5 individuals/concentration) after 21 days of BPA exposure was assessed by the comet assay under alkaline conditions following the Singh et al. [78] method. Using a small dissecting scissor, tissues were chopped into tiny pieces prior to being homogenized in a chilled buffer containing 0.075 M NaCl and 0.024 M Na2 EDTA. The cell suspension was centrifuged at 700 ×g for 10 min at 4 ^°^C, re-suspended twice in a chilled buffer, and finally, cell pellets were obtained. Cells were mixed with low-melting-point agarose and then spread over a frosted slide. After that, slides were immersed for ~ 15 min in a neutralization buffer, dried, and stained with ethidium bromide. The slides were visualized by an epi-fluorescence microscope (Leitz Orthoplan, Wetzlar, Germany) equipped with an excitation filter of 515–560 nm and a barrier filter of 590 nm. Randomly, ~ 100 cells per slide were selected to assess the comet cells. DNA damage was estimated as a percentage of tailed cells (%), tail length (µm), percentage of tail DNA (%), and tail moment (Arbitrary units) using a computer-based image analysis system (Comet Assay V software, Perspective Instruments).

### Histological examination of the digestive gland of *Ruditapes decussatus* clam

The digestive gland (*n* = 5 individuals/concentration) was carefully removed and kept in Davidson’s solution for 48 h, followed by transfer to 70% ethanol. Afterwards, tissues were processed in ascending concentrations of ethanol (80, 85, 90, 95, and 100%), infiltrated with xylene, and embedded in paraffin wax. Sections, 5 μm thick, were cut using a rotary microtome (Leica Reichert Jung Biocut 2030, USA), mounted on glass slides, stained with Harris hematoxylin, and counterstained with acidic eosin for histopathological examination. Slides were examined using light microscopy (Olympus BX41, Japan) and photomicrographed with a digital microscope camera (Lecia MC170 HD) to identify the histopathological changes in digestive gland tissues [48, 79].

### Semi-quantitative and quantitative histopathological analyses of the digestive gland of *Ruditapes decussatus* clams

For semi-quantitative analysis, the histopathological condition index was utilized based on the weighted indices approach stated by Joshy et al. [27], Costa et al. [48], and Cuevas et al. [49] to evaluate the health status of the *R. decussatus* clam’s digestive gland tissues. The histopathological condition index was estimated by the weight of each histopathological alteration (w_j_) **(**Table 1**)** and the degree of corresponding histopathological alteration (a_jh_) based on the following formula:

**Table 1:** The main histopathologies observed in the digestive gland of *Ruditapes decussatus* clams and their respective weights

Reaction pattern	Histopathological alteration	Weight
**Tubular alterations**	Vacuolation	1
	Tubular atrophy	2
	Necrosis	3
**Intertubular alterations**	Hemocyte infiltration	1
	Brown cells associated with lipofuscin-like pigments	1
	Fibrosis	2
	Necrosis	3

$${\text{I}}_{\text{h}}=\frac{{\sum}_{1}^{j}{{\text{w}}_{\text{j}}\text{a}}_{\text{j}\text{h}}}{{\sum\:}_{1}^{j}{\text{M}}_{\text{j}}}$$

Where I_h_: is the histopathological condition index for the individual h, w_j_: is the weight of the j^th^ histopathological alteration, a_jh_: is the score attributed to the h individual for the j^th^ alteration, M_j_: is the attributable maximum value for the j^th^ alteration (weight multiplied by the maximum score), and j: is the number of classified histopathological alterations in tissue. The weight value of the histopathological alteration ranges between 1 (minimum severity) and 3 (maximum severity) depending on its biological significance, and the score values are 0 (absent), 2 (infrequent), 4 (frequent), and 6 (diffuse). The denominator of the equation standardizes the value of the histopathological indices between 0 and 1 to allow comparisons between different groups [48]. The overall mean of the histopathological condition indices for five replicates from randomly selected areas of digestive gland sections per concentration (*n* = 5 replicates/concentration) was calculated and was classified as low (0.00–0.30), moderate (0.31–0.60), and high (0.61–1.0), according to Joshy et al. [27]. The precision of the histopathological analyses was verified by a series of blind reviews.

For quantitative analysis, the histomorphometry of the lumen and epithelial areas of the digestive gland tubules was analyzed through Image J software [80]. Different measurements from digestive gland tubular profiles were obtained from the average of five randomly selected digestive gland tubules of each animal’s section used per concentration (*n* = 5 individuals/concentration). These measurements include digestive tubule area (µm^2^), luminal area (µm^2^), and digestive tubule wall area (µm^2^), according to Tybinka et al. [81]. To indicate the changes in the digestive gland morphometry, both relative digestive tubule lumen area (%) and relative digestive tubule wall area (%) were calculated according to this equation:

$$\begin{aligned} & {\text{Relative digestive tubule lumen and}}/{\text{or wall area}} (\%) \\& \quad = \frac{{\text{Luminal and/or wall area}}\ (\mu {\text{m}}^2)}{{\text{Digestive tubule area}}\ (\mu {\text{m}}^2)} \times 100 \\\end{aligned}$$

### Statistical analysis of data

All results were presented as means of three (for the biochemical analyses) or five (for the comet assay and the semi-quantitative and quantitative histopathological analyses) independent samples ± standard errors (SE). All analyses were conducted using the free statistical computing and graphics software R (version 4.3.1, R Core Team [82]). A Kaplan-Meier estimator of survival probability was calculated for each treatment to analyze clam survival status. A log-rank test was performed for relevant survival and treatment combinations. Probabilities were estimated at each time point when an event occurred, and the products of those probabilities were used to estimate the survival proportions. Clam mortality was defined as an ‘event’, and clams that survived to the end of the experiment were considered ‘censored’ data. Since non-parametric survival analyses cannot explicitly incorporate information from replication (such as differences in survival among replicate aquaria), and due to the small size of the experiment, clams were pooled across replicate aquaria to estimate overall survival curves for each treatment (i.e., *n* = 40 individuals/concentration).

Analyses were performed using the “survival” package [83], while the “survminer” package was used for plotting the survival curves [84].

Biochemical markers, DNA damage parameters, and semi-quantitative and quantitative histopathological parameters were statistically analyzed using one-way analysis of variance (ANOVA), followed by Tukey’s honest significant difference (Tukey HSD) test. A significance level was set at *p* ≤ 0.05 for all analyses. Prior to the analysis, the normality of distributions was checked using the Shapiro-Wilk test, and the homogeneity of variances was explored by the Fligner-Killeen test and by a detailed visual inspection of the residuals’ plots. Package “ggplot2” was used to produce the final plots [85].

## Results

### Survival of *Ruditapes decussatus* clam

**Fig. 2:**
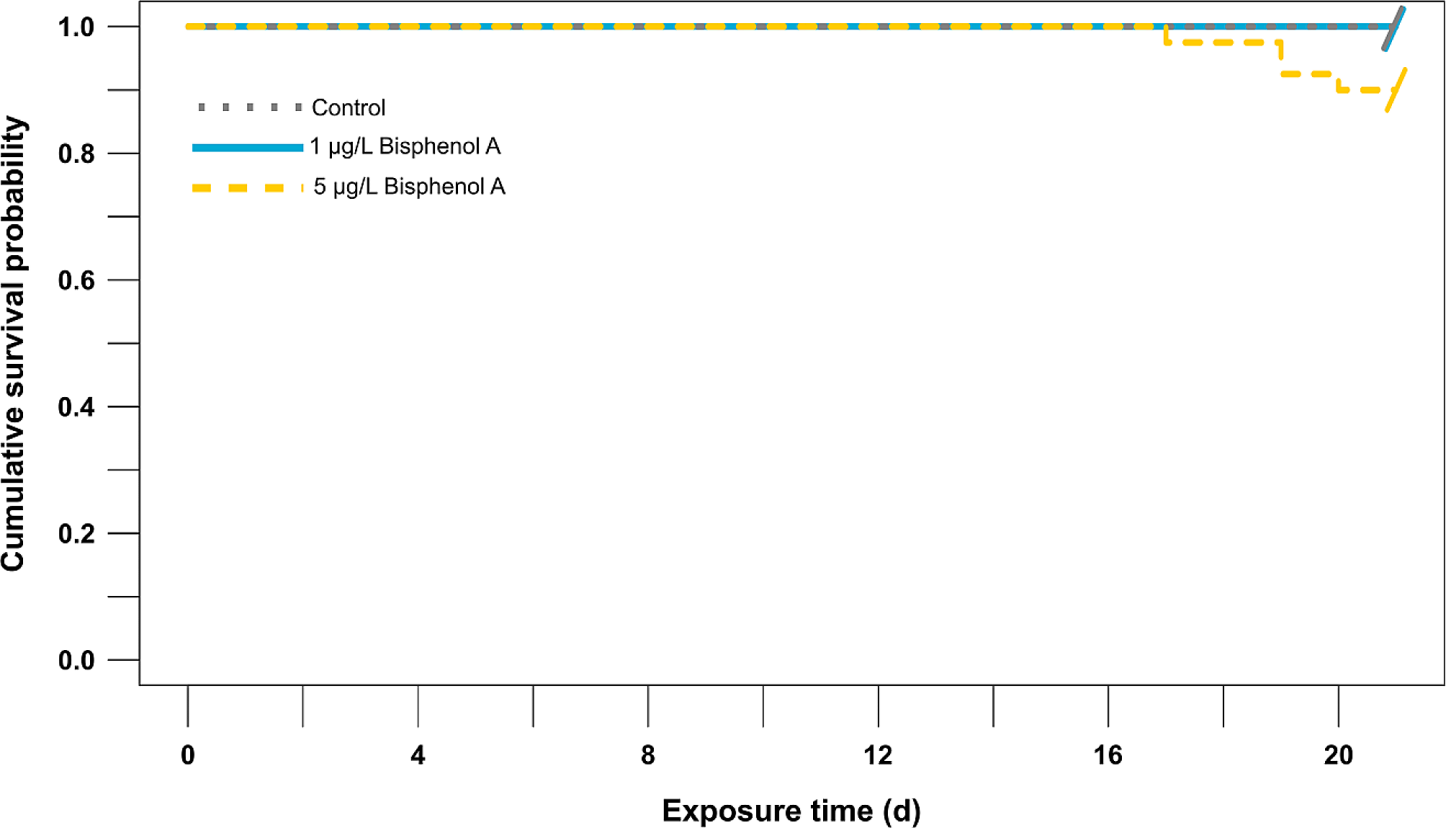
Kaplan-Meier survival curves showing cumulative survival probabilities of *Ruditapes decussatus* exposed to bisphenol A (1 µg/L “blue, solid line” and 5 µg/L “yellow, dashed line”) in comparing with a control group “grey, dotted line” for a period of 21 days. Marks (/) indicate censored data, i.e., clams at the respective treatment levels survived till the end of the experiment (pooled analysis, *n* = 40 individuals/concentration)

*R. decussatus* showed 100% survival under control laboratory conditions and after 21 days of exposure to 1 µg/L of BPA. However, 10% of the clam mortality was recorded during the experiment period in response to the 5 µg/L of BPA exposure (i.e., 4 clams died by the end of the experiment). Survival probabilities showed significant differences between treatments (log-rank test: χ^2^ = 8.3, df = 2, *p* = 0.02). With this so low mortality at 5 µg/L of BPA concentration, the medium time to death could not be calculated. No mortality occurred until the 17th day of the exposure period, when one clam died, followed by the death of two and one more individual on days 19 and 20, respectively **(**Fig. 2**)**.

### Biochemical marker responses of *Ruditapes decussatus* clam exposed to BPA

**Fig. 3:**
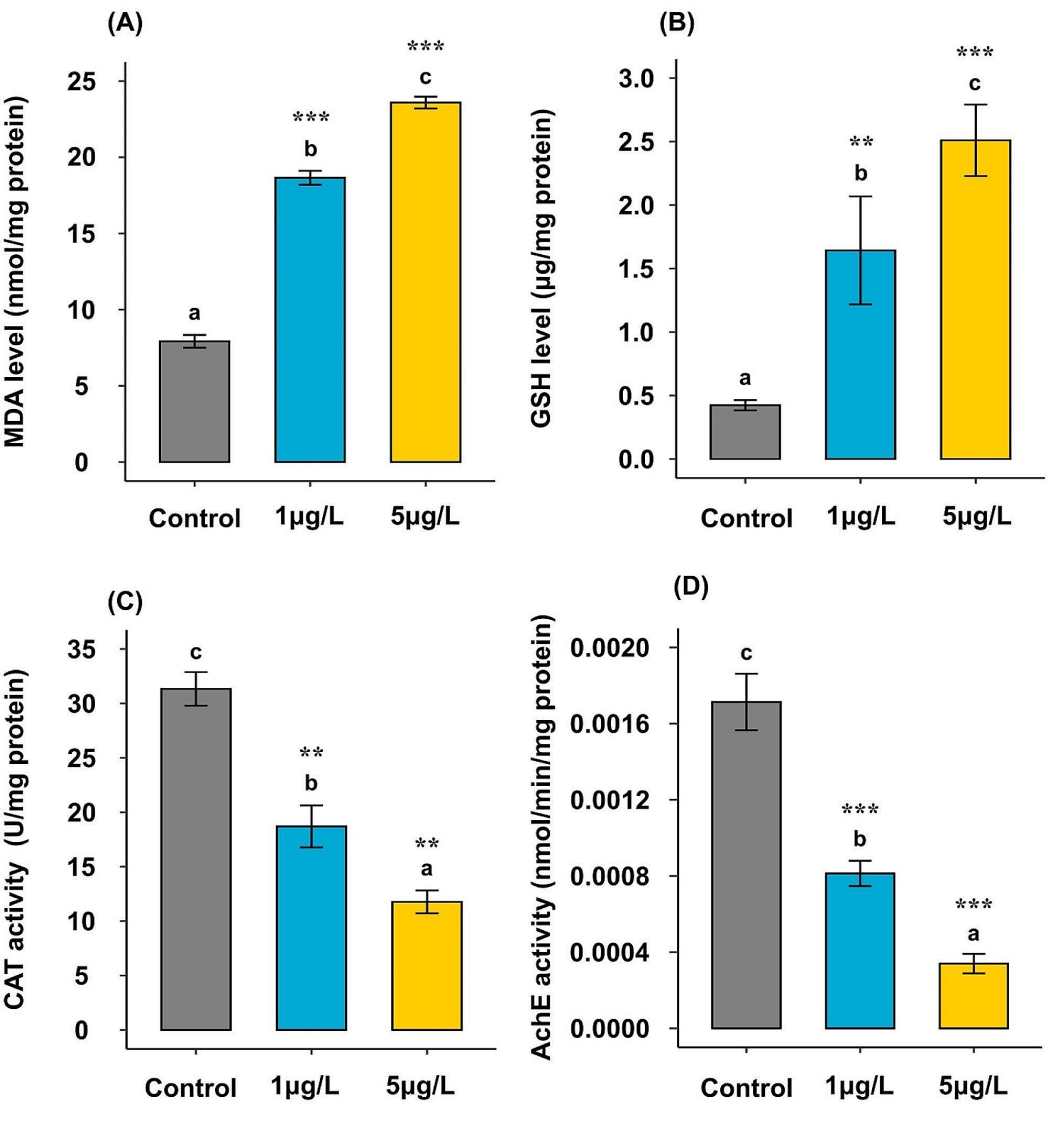
Average of biomarker responses estimated in *Ruditapes decussatus* digestive gland after exposure to water-borne bisphenol A (1 and 5 µg/L) for 21 days in comparing with a control group. **(A)** malondialdehyde (MDA) level (nmol/mg protein), **(B)** reduced glutathione (GSH) level (µg/mg protein), **(C)** catalase (CAT) activity (U/mg protein), and **(D)** acetylcholinesterase (AChE) activity (nmol/min/mg protein). Values are represented as mean ± SE; *n* = 3. Different letters ^(a, b, and c)^ indicate significant variations (*p* ≤ 0.05) among groups based on a one-way ANOVA test, followed by Tukey’s HSD test. Treatments vs. control group: * *p* ≤ 0.05, ** *p* ≤ 0.01, and *** *p* ≤ 0.001

The chronic impacts of BPA on the oxidative stress (MDA) and antioxidant (GSH and CAT) biomarkers in the *R. decussatus* digestive gland were investigated and summarized in Table 2; Fig. 3A-C. The results showed that the chronic effect of BPA yielded highly significant differences (*p* < 0.001) between treatments in the three measured parameters **(**Table 2**)**. The present results revealed significant increases (*p* < 0.001 and *p* < 0.01) in MDA and GSH levels, respectively, in animals subjected to 1 µg/L of BPA and highly significant increases (*p* < 0.001) in MDA and GSH levels in animals exposed to 5 µg/L of BPA in comparison to the control group **(**Fig. 3A**&B)**. The MDA level increased in the *R. decussatus* digestive gland by change percentages of 135 and 197% after exposure to 1 and 5 µg/L of BPA, respectively, compared to the control group **(**Fig. 3A**)**. At the same time, the GSH level was more highly induced in clams subjected to 1 and 5 µg/L of BPA, showing an increasing percentage of about 290 and 498%, respectively, compared to the control group **(**Fig. 3B**).** Additionally, highly statistical significant differences (*p* < 0.001) in MDA level between the two treatment groups of BPA (1 and 5 µg/L) were recorded **(**Fig. 3A**)** and a lesser degree of significant difference (*p* = 0.02) in GSH level between them **(**Fig. 3B).

**Table 2:** Statistical results of a one-way analysis of variance (ANOVA) of the biochemical parameters measured in *Ruditapes decussatus* digestive glands following exposure to bisphenol A (1 and 5 µg/L) concentrations for the experimental time of 21 days in comparing with a control group

Parameter	Source of variation	df		SS	MSS	F-value	*p*-value
MDA (nmol/mg protein)	Between groups	2	385.1		192.56	359	**< 0.001**
	Within groups	6	3.2		0.54		
	Total	8	388.3				
GSH (µg/mg protein)	Between groups	2	6.594		3.297	38.55	**< 0.001**
	Within groups	6	0.513		0.086		
	Total	8	7.107				
CAT (U/mg protein)	Between groups	2	590.5		295.26	40.94	**< 0.001**
	Within groups	6	43.3		7.21		
	Total	8	633.8				
AChE (nmol/min/mg protein)	Between groups	2	2.920 × 10^− 6^		1.46 × 10^− 6^	50.25	**< 0.001**
	Within groups	6	1.743 × 10^− 7^		2.91 × 10^− 6^		
	Total	8	3.09 × 10^− 6^				

All assumptions (e.g., normality, etc.) for statistical methods were met. Significant results (*p* ≤ 0.05) are highlighted in **bold**. df: degree of freedom, SS: sum of squares, MSS: mean sum of squares, F-value: Fisher’s function, and *p*-value: significance level

On the contrary, the CAT activity showed significant statistical reductions (*p* < 0.01) in two BPA treatments (1 and 5 µg/L) compared to the control group (Fig. 3C**)**. The CAT depletion levels revealed a concentration-dependent manner, where CAT levels decreased by about 40.25 and 62.42% in the *R. decussatus* digestive gland exposed to 1 and 5 µg/L of BPA, respectively, compared to the control group. The statistically significant difference (*p* = 0.04) in CAT level was recorded between the two treatment groups of BPA (1 and 5 µg/L) **(**Fig. 3C**)**.

The neurotoxic impacts induced by the BPA in the digestive gland of *R. decussatus* were evaluated by determining the AChE activity **(**Fig. 3D**)**. The highly significant variation (*p* < 0.001) in AChE activity was determined among all groups **(**Table 2**)**. Highly significant inhibition (*p* = 0.001) in the AChE activity with 52% reduction was recorded in animals after exposure to 1 µg/L of BPA compared to the control group. However, exposure to 5 µg/L of BPA resulted in the most dramatic inhibition (*p* < 0.001), with an 80% reduction in the AChE activity compared to the control group. Moreover, a marginal statistical reduction (*p* = 0.03) in the AChE activity was recorded between the two BPA treatments (1 and 5 µg/L) **(**Fig. 3D**)**.

### Estimation of DNA damage in the digestive gland of *Ruditapes decussatus* clam by comet assay

**Fig. 4:**
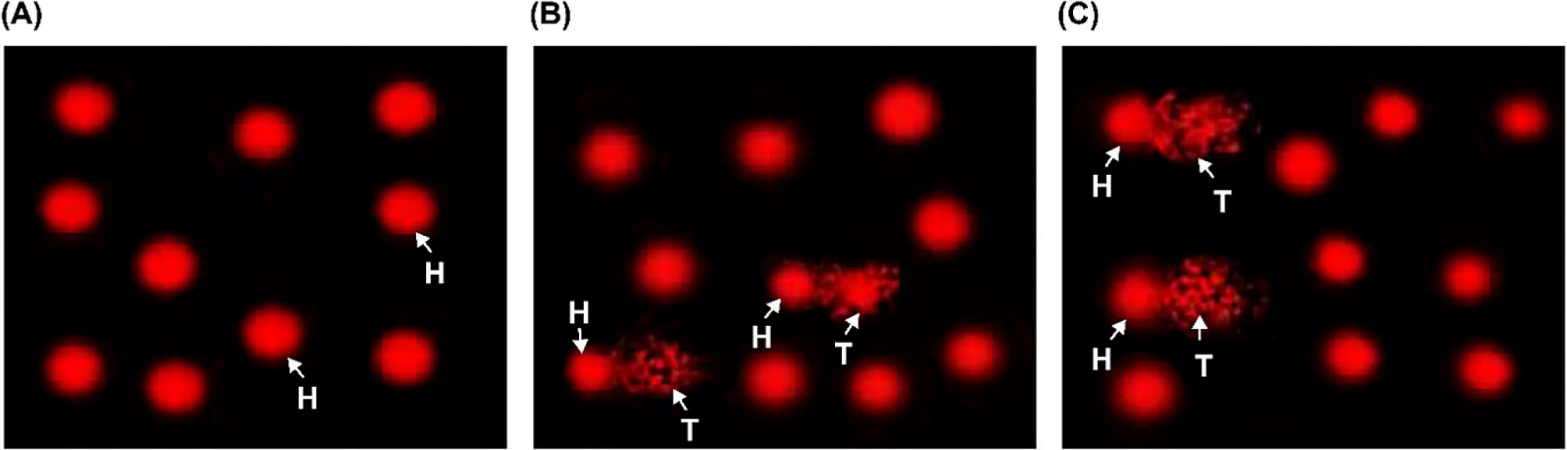
Photomicrographs of comet assay (DNA damage) in *Ruditapes decussatus* cells isolated from digestive gland tissues following exposure to different bisphenol A concentrations (1 and 5 µg/L) for a period of 21 days of experimental time comparing with a control group. **(A)** control, **(B)** 1 µg/L, and **(C)** 5 µg/L. Abbreviations: H: head and T: tail

To evaluate DNA integrity in the *R. decussatus* digestive gland cells after 21 days of BPA exposure, the present study conducted a comet assay. BPA instigated genotoxic consequences in *R. decussatus* digestive glands **(**Fig. 4A-C**)**. A visual examination of microphotographs of comets clearly revealed that the DNA molecules extracted from *R. decussatus* digestive gland cells of the control group did not exhibit detrimental alterations. Instead, they exhibited a symmetrical bright nucleus surrounded by a thin halo **(**Fig. 4A**)**. Conversely, the DNA of the clam groups subjected to concentrations of 1 and 5 µg/L exhibited a noticeable ‘comet’ formation after electrophoresis due to breakdown and migration of fragments of genomic DNA **(**Fig. 4B**&C)**.

**Fig. 5:**
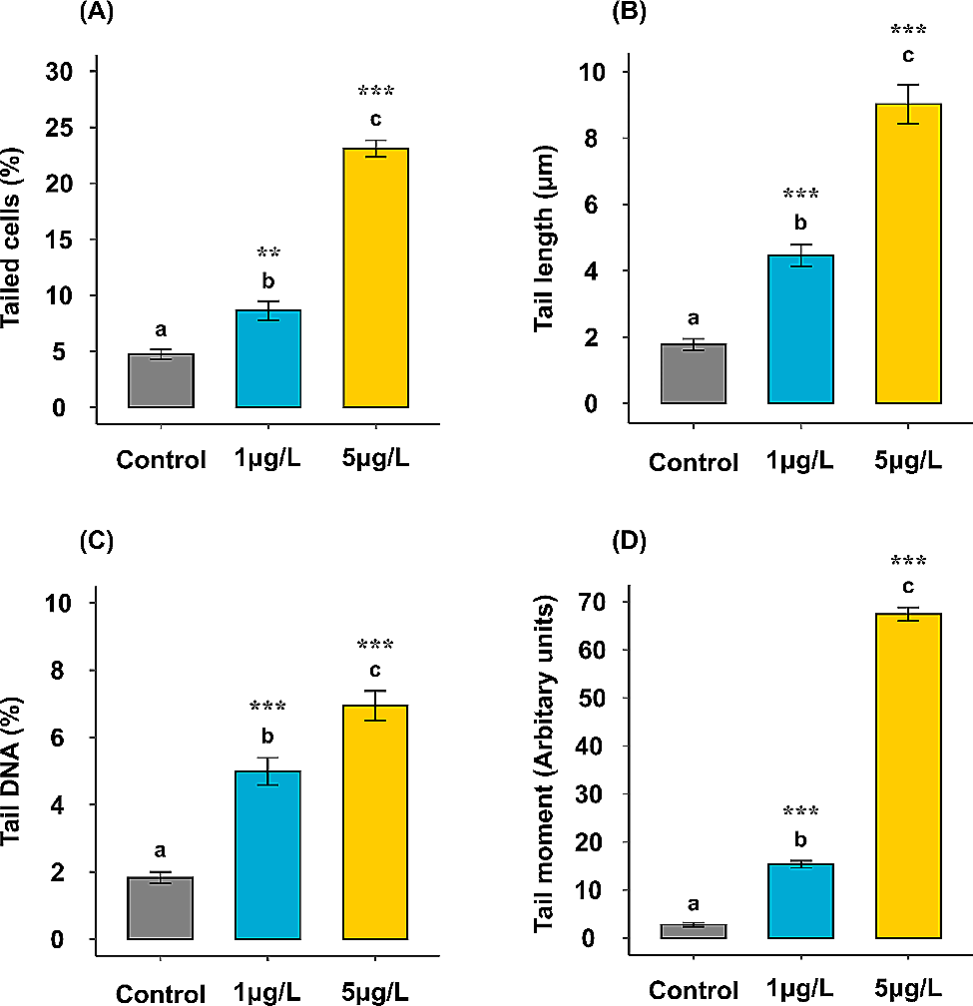
Average DNA damage by comet assay measured in *Ruditapes decussatus* cells isolated from digestive gland tissues after 21 days of exposure to water-borne bisphenol A (1 and 5 µg/L) in comparing with a control group. **(A)** percentage of tailed cells (%), **(B)** tail length (µm), **(C)** DNA percentage in the comet tail (%), and **(D)** tail moment (Arbitrary units). Values are represented as mean ± SE; *n* = 5. Different letters ^(a, b, and c)^ indicate significant variations (*p* ≤ 0.05) among groups based on a one-way ANOVA test, followed by Tukey’s HSD test. Treatments vs. control group: * *p* ≤ 0.05, ** *p* ≤ 0.01, and *** *p* ≤ 0.001

Data represented in Table 3 illustrate the highly significant differences (*p* < 0.001) among all groups in the four measured parameters, including the percentage of tailed cells, the length of the comet tail, the percentage of DNA in the comet tail, and the tail moment. In general, the control group showed slightly less than 5% of nuclei with DNA damage, while the mean percentages of the digestive gland cells with damaged DNA from the groups treated with 1 and 5 µg/L of BPA had about two (*p* = 0.005) and five times (*p*˂ 0.001), respectively, higher than that recorded in the control group. A highly significant variation (*p*˂ 0.001) was recorded between two treatment groups (1 and 5 µg/L) **(**Fig. 5A**)**. Specifically, there were highly significant variations (*p* ≤ 0.001) in the digestive gland tissues of *R. decussatus* with DNA impairment, including length of comet tail, DNA percentage in comet tail, tail moment in the treated groups with 1 and 5 µg/L of BPA compared with a control group. At the same time, these parameters presented significant variations (*p*˂ 0.01) among the two treated groups (1 and 5 µg/L) **(**Fig. 5B-D**)**.

**Table 3:** Statistical results of a one-way analysis of variance (ANOVA) of DNA damage parameters in *Ruditapes decussatus* digestive glands following exposure to bisphenol A (1 and 5 µg/L) concentrations for the experimental time of 21 days in comparing with a control group

Parameter	Source of variation	df	SS	MSS	F-value	*p*-value
Tailed cells (%)	Between groups	2	934.9	467.5	191.6	**< 0.001**
	Within groups	12	29.3	2.4		
	Total	14	964.2			
Tail length (µm)	Between groups	2	134.48	67.24	83.59	**< 0.001**
	Within groups	12	9.65	0.8		
	Total	14	144.13			
Tail DNA (%)	Between groups	2	66.6	33.3	51.75	**< 0.001**
	Within groups	12	7.72	0.64		
	Total	14	74.32			
Tail moment (Arbitrary units)	Between groups	2	11,755	5878	1317	**< 0.001**
	Within groups	12	54	4		
	Total	14	11,809			

All assumptions (e.g., normality, etc.) for statistical methods were met. Significant results (*p* ≤ 0.05) are highlighted in **bold**. df: degree of freedom, SS: sum of squares, MSS: mean sum of squares, F-value: Fisher’s function, and *p*-value: significance level

### Histological alterations assessment of *Ruditapes decussatus* clam digestive gland

**Fig. 6:**
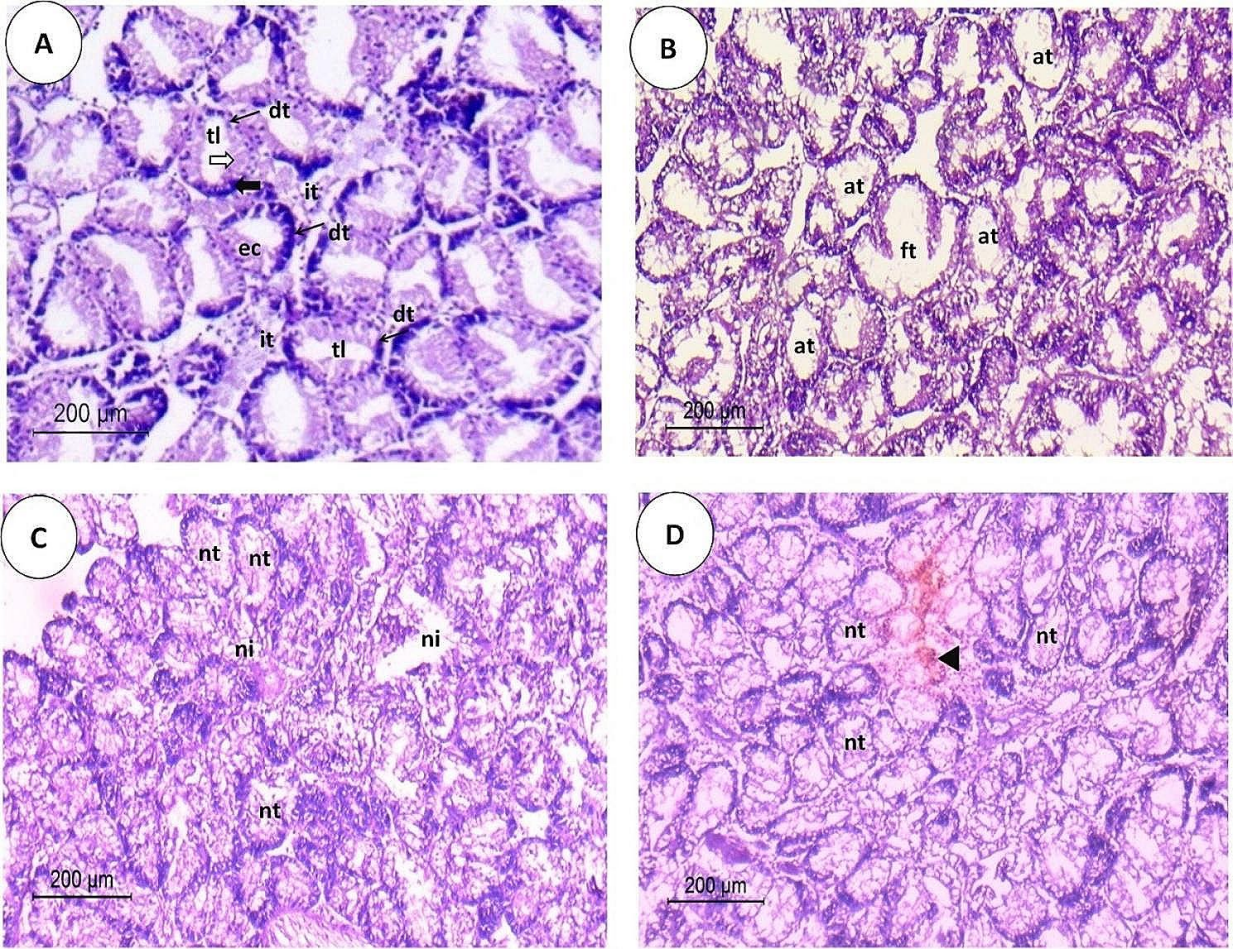
Light photomicrographs of transverse sections, 5 μm in thickness and stained with H&E, through the *Ruditapes decussatus* digestive glands showing histopathological alterations in the digestive gland structure after exposure to different concentrations of bisphenol A (BPA) for 21 days in comparing with the normal structure of a control group. **(A)** Digestive glands of the control group showing normal structure with digestive tubules that are composed of a single layer of digestive **(white wide arrow)** and basophilic **(black wide arrow)** epithelial cells surrounding a narrow tubular lumen and normal intertubular connective tissue connecting these tubules, **(B)** Digestive glands subjected to 1 µg/L of BPA showing fused and atrophied digestive tubules represented by a diminution in the thickness of the epithelial cell layer and widening of the tubular lumen, and **(C-D)** Digestive glands subjected to 5 µg/L of BPA showing necrotic tubules, necrotic intertubular connective tissue, and the presence of lipofuscin pigment **(black arrowhead)**. **(dt)** digestive tubule, **(ec)** epithelial cells, **(tl)** tubule lumen, **(it)** intertubular tissue, **(ft)** fused tubules, **(at)** atrophied tubules, **(nt)** necrotic tubules, and **(ni)** necrotic intertubular tissue

**Fig. 7:**
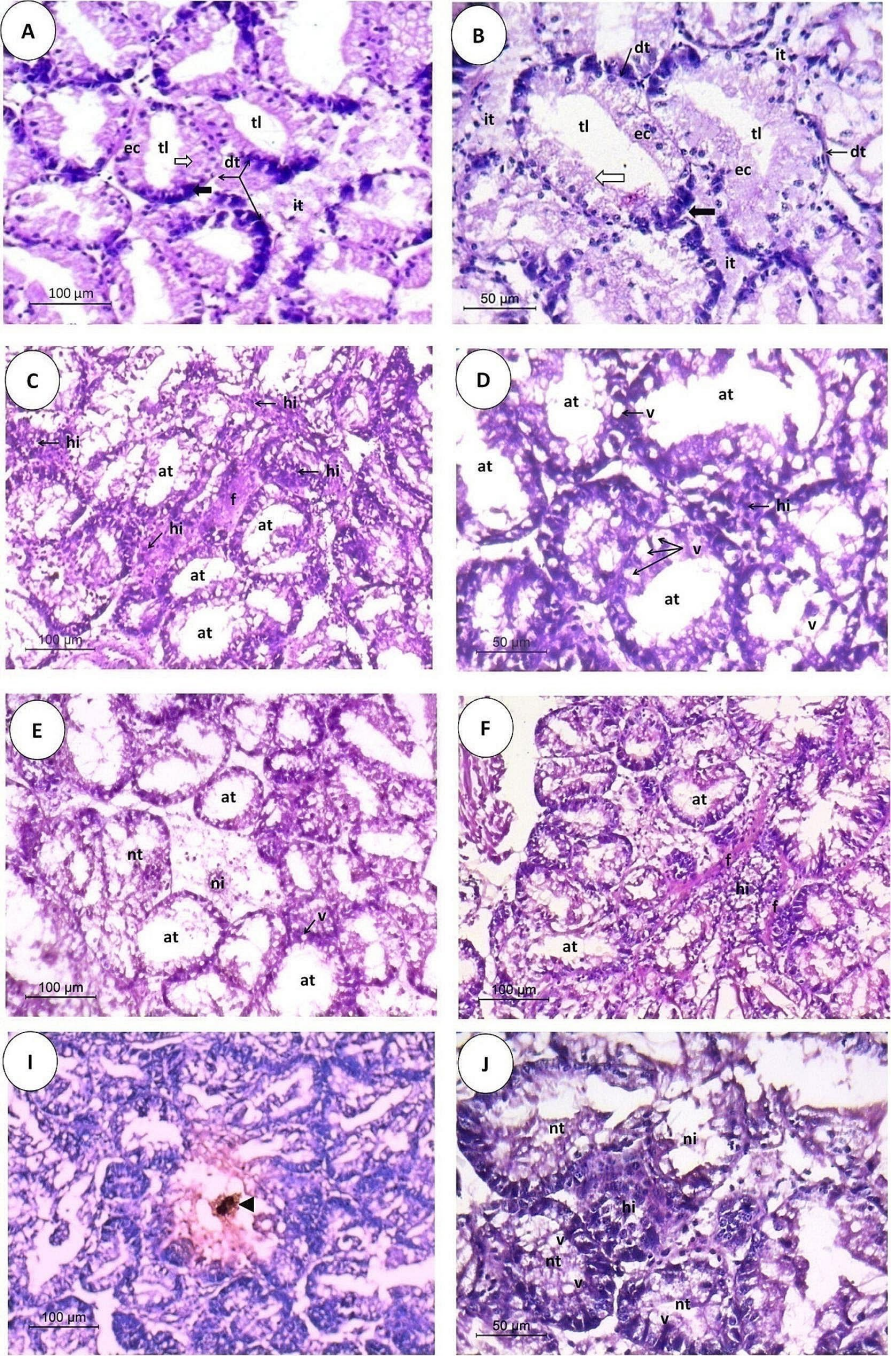
Light photomicrographs of transverse sections, 5 μm in thickness and stained with H&E, through the *Ruditapes decussatus* digestive glands showing histopathological alterations in the digestive gland structure after exposure to different concentrations of bisphenol A (BPA) for 21 days in comparing with the normal structure of a control group. **(A-B)** Digestive glands of the control group showing normal structure with digestive tubules that are composed of a single layer of digestive **(white wide arrow)** and basophilic **(black wide arrow)** epithelial cells, surrounding a narrow tubular lumen, and normal intertubular connective tissue connecting these tubules, **(C-D)** Digestive glands subjected to 1 µg/L of BPA showing atrophied digestive tubules, represented by a diminution in the thickness of the epithelial cell layer and widening of the tubular lumen, hemocyte infiltration around the damaged digestive gland tubules, fibrosis in the intertubular connective tissue, and tubular vacuolation with disorganization of the lining epithelium, and **(E-J)** Digestive glands subjected to 5 µg/L of BPA showing atrophied and necrotic tubules and necrotic intertubular connective tissue with heavy hemocyte infiltration, especially granulocytes, tubular vacuolation, and the presence of brown cells associated with lipofuscin-like pigments **(black arrowhead)**. **(dt)** digestive tubule, **(ec)** epithelial cells, **(tl)** tubule lumen, **(it)** intertubular tissue, **(at)** atrophied tubules, **(hi)** hemocyte infiltration, **(f)** fibrosis, **(v)** vacuoles, **(nt)** necrotic tubules, and **(ni)** necrotic intertubular tissue

The histology of the digestive gland tissues of control and treated *R. decussatus* clams with BPA is presented in Figs. 6 and 7. Unexposed control *R. decussatus* clams showed the normal histological structure of the digestive gland, consisting of intact digestive tubules constituted by a single layer of epithelial cells of two main cell types: digestive and basophilic cells surrounding a narrow tubular lumen. Digestive cells are characterized by their columnar shape and small basal nuclei, while basophilic cells have a triangular shape and large nuclei. The normal intertubular connective tissue mainly consists of a low number of fibrocytes and a normal distribution of hemocytes **(**Figs. 6A and 7A-B**)**. Exposure to different BPA concentrations (1 and 5 µg/L) for 21 days apparently damaged the structure of the *R. decussatus* digestive gland. The digestive glands subjected to 1 µg/L of BPA represented histological alterations, including a fusion in atrophic digestive gland tubules characterized mainly by a narrowing of the tubular lining with a low cuboidal appearance and dilation of the tubular lumen **(**Fig. 6B**)**. Vacuolization in the digestive epithelial cells and the appearance of fibrosis and hemocyte infiltration, typically granulocytes in the damaged intertubular connective tissue, were the most commonly noticed histological alterations in the digestive glands subjected to 1 µg/L of BPA **(**Fig. 7C-D**)**.

At the same time, severe lesions were recorded in digestive glands subjected to 5 µg/L of BPA. Atrophic and necrotic digestive tubules were mostly associated with a detachment of some epithelial cells within the digestive tubules’ lumen, resulting in occlusion of the lumen. Hemocyte infiltration and brown cells associated with lipofuscin-like pigments were observed in the intertubular connective tissue among the damaged digestive gland tubules, implying inflammatory responses. The vacuolation within the epithelial cells and intertubular fibrosis were also recorded. All these alterations destroy the digestive tubules of *R. decussatus* subjected to 5 µg/L of BPA **(**Figs. 6C-D and 7E-J**)**.

### Semi-quantitative and quantitative histopathological analyses of the digestive gland of *Ruditapes decussatus* clams

**Fig. 8:**
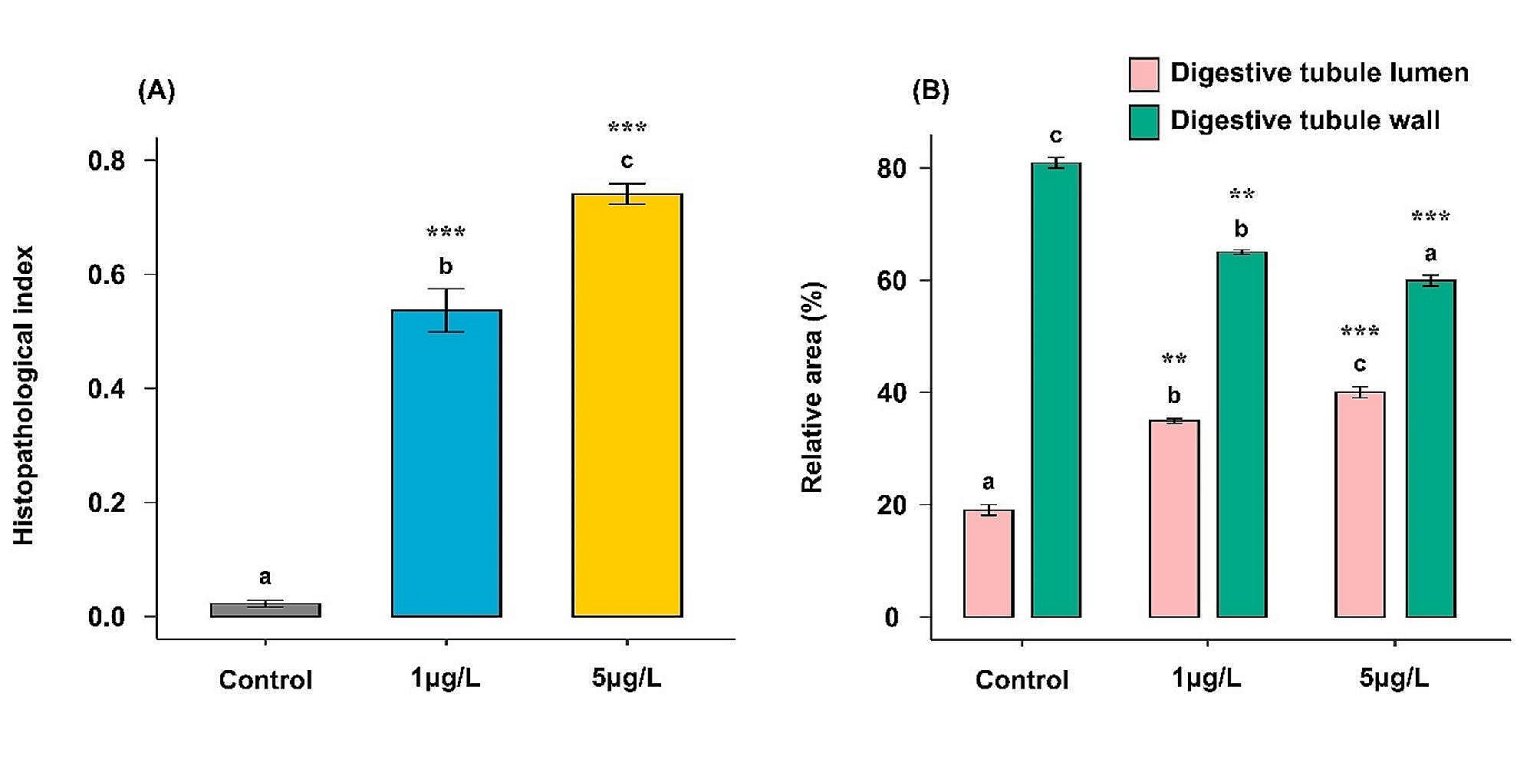
Semi-quantitative and quantitative analyses of the histopathological alterations of the digestive gland of *Ruditapes decussatus* clams after exposure to water-borne bisphenol A (1 and 5 µg/L) for 21 days in comparing with a control group. **(A)** The semi-quantitative histopathological condition indices (I_h_) of the digestive gland and **(B)** Histomorphometric changes of the lumen and epithelial areas of the digestive gland tubules. Values are represented as mean ± SE; *n* = 5. Different letters ^(a, b, and c)^ indicate significant variations (*p* ≤ 0.05) among groups of the same parameter based on a one-way ANOVA test, followed by Tukey’s HSD test. Treatments vs. control group: * *p* ≤ 0.05, ** *p* ≤ 0.01, and *** *p* ≤ 0.001

The analysis of the histopathological condition indices of the digestive gland of *R. decussatus* clams revealed that there was a significant variation (*p* < 0.001) between groups **(**Table 4**)**. Exposure of clams to different BPA concentrations (1 and 5 µg/L) for 21 days showed significant differences (*p* < 0.001) compared with the control clams. The clams subjected to BPA with a 5 µg/L concentration attained the highest value of histopathological condition index (0.71 ± 0.01, high I_h_), which was significantly different (*p* ≤ 0.001) from other clam groups, followed by clams subjected to BPA with a 1 µg/L concentration (0.53 ± 0.03, moderate I_h_) and control clams (0.02 ± 0.005, low I_h_) **(**Fig. 8A**)**.

**Table 4:** Statistical results of a one-way analysis of variance (ANOVA) of histopathological semi-quantitative and quantitative parameters in *Ruditapes decussatus* digestive glands following exposure to bisphenol A (1 and 5 µg/L) concentrations for the experimental time of 21 days in comparing with a control group

Parameter	Source of variation	df	SS	MSS	F-value	*p*-value
Histopathological index	Between groups	2	1.291	0.645	217.383	**< 0.001**
	Within groups	12	0.036	0.003		
	Total	14	1.326			
Relative digestive tubule lumen and/or wall area (%)	Between groups	2	1196.0	598	180.1	**< 0.001**
	Within groups	12	39.9	3.3		
	Total	14	11,999			

All assumptions (e.g., normality, etc.) for statistical methods were met. Significant results (*p* ≤ 0.05) are highlighted in **bold**. df: degree of freedom, SS: sum of squares, MSS: mean sum of squares, F-value: Fisher’s function, and *p*-value: significance level

The histomorphometric data displayed in Fig. 8B indicate that there was a significant variation (*p* < 0.001) between groups **(**Table 4**)**. The morphometric analysis of the digestive gland tubules showed that the two BPA-treated groups (1 and 5 µg/L) were atrophied in a concentration-dependent manner through significant increases (*p* = 0.002 and *p* < 0.001 for low and high concentrations, respectively) of the values of the relative digestive tubule lumen area and significant decreases (*p* = 0.002 and *p* < 0.001 for low and high concentrations, respectively) of the values of the relative digestive tubule wall area as compared with the control group.

## Discussion

BPA is a highly prevalent environmental contaminant and hormonal disruptor [21]. The presence of BPA in aquatic ecosystems can cause harmful consequences for creatures at several biological levels [86, 87]. The present study was designed to assess the potential impact of chronic exposure to 1 and 5 µg/L of BPA on *R. decussatus* clam health. The duration of BPA exposure in this study was 21 days, which allowed enough time to manifest the impacts of harmful substances in clams [62, 88].

In the current work, the clam *R. decussatus* showed 100% survival in the control treatment and response to a low dose of BPA (1 µg/L) after 21-day exposure. Low mortality (10%) was only observed at 5 µg/L of BPA. Similarly, as a result of the BPA genotoxic effect, freshwater invertebrates like *Daphnia magna* and *Chironomus riparius* demonstrated a reduction in survival rate after exposure to 0.3 µg/L and 500 µg/L of BPA, respectively, for a period of 24 h [89]. Moreover, the blood clam *Tegillarca granosa* showed a mortality rate of ~ 24% after 2 weeks of exposure to 100 ng/L of BPA [90]. Survival impairment at a high BPA concentration in this study might be considered as a consequence of serious progress of level toxicities that are discussed below in detail.

An extensively recognized mechanism of BPA’s toxic effect in aquatic animals is its capacity to induce oxidative stress damage by producing elevated levels of intracellular reactive oxygen species (ROS) interacting with biomolecules, such as cellular lipids, carbohydrates, proteins, and DNA [16, 17, 89]. This interaction results in diverse cellular and tissue impairments in aquatic bivalve species, including mitochondrial dysfunction and the formation of lipid peroxidation, resulting in a decrease in cell viability [16, 91]. Also, BPA can impair the natural defense system of these organisms, including endogenous enzymatic and non-enzymatic antioxidants involved in the maintenance of tissue redox balance, and induce adverse effects by inhibiting antioxidant enzyme activities [19, 92, 93].

The current results showed that the chronic impact of BPA revealed significant elevations in MDA levels in the *R. decussatus* digestive gland subjected to 1 and 5 µg/L of BPA when compared to the control group, and these increases exhibited a concentration-dependent manner. The significant increase in MDA levels indicated that BPA induced oxidative stress and lipid oxidation damage in the *R. decussatus* digestive gland. Hence, the excessive production of cellular ROS can lead to the oxidation of unsaturated fatty acids in membrane lipids through radical reactions. This process causes alterations in membrane permeability and fluidity, in addition to the accumulation of oxidized lipid products like MDA, which is commonly used as a reliable biomarker to assess the extent of membrane lipid peroxidation in the presence of BPA pollution [94, 95]. This result is consistent with the previous findings in bivalves subjected to different levels of BPA concentration [16, 93, 96]. Moreover, Jenzri et al. [17] observed a significant elevation in MDA levels in the tissues of the respiratory tree and digestive tube of the sea cucumber *Holothuria poli* exposed to BPA.

Herein, the elevated stimulation of GSH levels in the *R. decussatus* digestive gland by BPA is an attempt to mitigate the harmful oxidative stress resulting from the excessive ROS produced during BPA metabolization. According to Abd Elkader and Al-Shami [16], the rise in GSH levels is probably due to an enhancement in antioxidant defenses caused by an increase in ROS production generated by BPA. This finding aligns with the findings of Jenzri et al. [17], who recorded a notably elevated GSH level in the respiratory tree and digestive tube tissues of sea cucumbers subjected to BPA compared to the control group.

Even though the level of GSH increased in the *R. decussatus* digestive gland exposed to 1 and 5 µg/L of BPA, the level of MDA remained high, indicating the high toxicity of BPA and the antioxidant capacity of *R. decussatus* clams was inadequate to eliminate the ROS produced during the metabolism of BPA efficiently and to prevent the damage caused by lipid oxidation. Therefore, the present results recorded significant reductions of CAT activity in the *R. decussatus* digestive gland exposed to 1 and 5 µg/L of BPA when compared to the control group in a concentration-dependent manner. This reduction emphasizes the difficulty in countering and neutralizing ROS induced by BPA, causing damage to clam tissues and reducing the clam’s ability to produce antioxidants that mitigate the toxicity [28, 97].

In the current work, the neurotoxicity of BPA on the *R. decussatus* digestive gland was evaluated by quantifying the AChE activity in order to investigate its connection with oxidative stress. Herein, a highly significant inhibition in the AChE activity was reported, with 52 and 80% reduction percentages in clams subjected to 1 and 5 µg/L of BPA, respectively, as compared to the control group. This finding is corroborated by prior studies conducted on several organisms, which observed a decrease in AChE activity when subjected to BPA [17, 81, 98, 99]. The neurotoxic effects of BPA on AChE can be explained by its interactions with amino acids in the enzyme’s active site, mainly in the peripheral anionic binding site and acyl binding site, as revealed in a previous study by Yilmaz et al. [100]. The inhibition of AChE by neurotoxic bisphenols caused inhibition in ACh hydrolysis, resulting in an overabundance of ACh, which in turn stimulates cholinergic receptors and disrupts nerve function [101, 102].

BPA-induced DNA change in the clam digestive glands was evidenced in this study by a significant increase in the percentage of tailed cells, tail length, tail DNA percentage, and tail moment, with BPA concentration-dependent.

Our findings are in complete agreement with the earlier works on aquatic invertebrates, for example, the genotoxicity potential of BPA that was detected in the somatic cells of *D. magna* and *C. riparius* [89], the sperm cells and hemocytes of the amphipods *Echinogammarus veneris* and *Gammarus aequicauda* [103], and the adductor muscles of the bivalve *Lithophaga lithophaga* [16]. Dose-dependent DNA damage might be related to a surplus of ROS production, causing malfunction of the antioxidant defense system [60]. BPA enhances redox imbalance and participates in the free radical cascade, which is likely the reason for DNA impairments [16, 89].

Due to its lipophilic nature, BPA has the ability to accumulate in the digestive gland, making it a possible organ susceptible to the damaging effects of BPA. The high sensitivity of epithelial cells lining the digestive gland tubules to the deleterious impacts of numerous marine contaminants is directly attributed to their role in metabolism as well as the detoxification process [104]. Herein, the histological examination of *R. decussatus* digestive gland subjected to 1 and 5 µg/L of BPA for 21 days revealed distinct histological alterations. Under exposure to 1 µg/L of BPA, hemocyte infiltration and fibrosis were observed in the damaged intertubular connective tissues, and the atrophied digestive tubules with vacuolization in the digestive epithelial cells were also recorded. BPA exposure induced an inflammatory response in the *R. decussatus* digestive gland through the mediation of the recruitment of hemocytes in damaged tissue. As hemocyte infiltration is the main internal immune defense of bivalves for detoxification, it has been associated with significant histopathological damage produced by hazardous substances [105, 106]. In bivalves, intertubular connective tissue fibrosis is a common pathological feature of digestive gland tissue under chronic exposure to pollutants. It occurs through the proliferation of fibroblasts inside connective tissue, destroying tissue structure and function. Thus, fibrosis is considered a good biomarker of environmental quality [48]. Digestive tubule atrophy is a commonly observed lesion in bivalves subjected to environmental contamination [48, 49]. It appears as a dilation of the tubular lumen and diminution of the tubular lining, leading to failure of digestive gland functions [107]. The vacuoles of digestive cells are normally connected to a system of heterophagosomes and lysosomes responsible for intracellular digestion [108–110]. However, the increase in vacuolization in digestive gland cells is related to a sudden increase in the lysosome volume in bivalves when exposed to xenobiotics of both organic and inorganic origins [111–113].

In addition to previous histological aberrations, severe lesions were recorded in digestive glands subjected to 5 µg/L of BPA, including the presence of necrotic digestive tubules and the presence of brown cells associated with lipofuscin-like pigments in the intertubular connective tissue. Exposure to harmful chemicals induces necrosis, eliciting a pro-inflammatory reaction in the neighbouring cells [114]. BPA was recorded to cause a necrotic pathway, as reported by Benjamin et al. [62], under continued exposure to 3 µg/L of BPA. The accumulation of lipofuscin granules, byproducts of lipid peroxidation consisting of lipids and proteins, in the digestive gland tissues of bivalves exposed to environmental contamination resulted from the alteration in the lysosomes of digestive gland epithelial cells [115]. It is a good indicator of tissue deterioration through the oxidation of polyunsaturated fatty acids in the digestive gland by free oxygen radicles [116–118].

The qualitative histopathological assessment with semi-quantitative and quantitative histopathological analyses presents valuable information for the health status of marine organisms [119, 120]. Therefore, this study used a histopathological condition index approach in the digestive gland of *R. decussatus* clams to enable the integration of all alterations into a single value to facilitate the comparison between the health statuses of clams and detect the extent and severity of histopathological changes after exposure to different concentrations of BPA. Based on the histopathological condition indices of the digestive gland, exposure of clams to different BPA concentrations (1 and 5 µg/L) for 21 days showed significant differences compared with the control clams. The highest value of the histopathological condition index was recorded in clams exposed to BPA of 5 µg/L concentration, which was significantly different from other clam groups, followed by clams subjected to BPA with 1 µg/L concentration (moderate I_h_) and control clams (low I_h_). These results indicate that the digestive gland of clams exposed to BPA of 5 µg/L concentration was more impacted than the digestive gland of clams exposed to BPA of 1 µg/L concentration. Consistently, previous studies on metals and nanoparticles’ impact on the digestive glands of *Mytilus galloprovincialis* revealed a similar histopathological condition index pattern, as high pollutant doses showed significant differences compared to the control group [119, 121].

The present study also used the morphometric analysis of the digestive gland tubules to detect the variations between groups in the areas of the digestive tubule lumen and wall. The present result revealed atrophy in tubules in the two BPA-treated groups (1 and 5 µg/L) in a concentration-dependent manner through significant increases in the values of the relative digestive tubule lumen area and significant decreases in the values of the relative digestive tubule wall area as compared with the control group. Overall, the present study indicated that chronic exposure of *R. decussatus* clams to BPA (1 and 5 µg/L) led to an increase in the prevalence of histopathological alterations in the digestive gland tissues compared to the control clams.

In line with the current results, Benjamin et al. [62] indicated similar histological responses like hemocyte infiltration in the intertubular spaces with tubular atrophy, vacuolations, necrosis, fibrosis, and lipofuscin aggregation in the tissues of *Corbicula fluminea* at different concentrations of BPA (1, 2, and 3 µg/L) under a 21-day exposure. Jenzri et al. [17] documented an inflammatory reaction characterized by a significant infiltration of dark nucleated cells in the sea cucumber respiratory tree subjected to BPA. Also, similar histological alterations have been recorded in the digestive glands of bivalves subjected to different environmental xenobiotics [27, 48, 49, 110, 122–125]. The variation in histological alterations proves that *R. decussatus* clams are good indicators of BPA. As histopathological lesions, including tubular atrophy, necrosis of digestive tubules and intertubular tissue, fibrosis, and inflammation, can potentially serve as biomarkers for assessing environmental quality [48].

## Conclusion

To summarize, the current results revealed the toxic effects of chronic BPA exposure on *R. decussatus.* 21-day treatment with BPA caused significant impairments to the clam digestive gland, which might be the basis of a series of subsequent body responses. Survival analysis showed that minimal mortality occurred only at an elevated BPA concentration (5 µg/L), emphasizing that the effect of BPA on clam survival was dose-dependent. Our study demonstrated that exposure to BPA impacted the *R. decussatus* antioxidant defense system due to its toxic effect. Furthermore, there is a significant reduction in AChE activity, indicating a disturbance in the cholinergic function. Genotoxicity and histological alterations in the digestive gland tissues were also detected. More in-depth, this work highlighted the importance of a multi-marker approach to draw a complete picture of the chemical toxicity of marine bivalves since each single biomarker offers different types of complementary information, which is a primary asset in this study type. Finally, in our holistic approach, the clam *R. decussatus* has shown its usefulness as a biomonitor species for detecting the impact of BPA pollution in marine ecosystems.

## Data Availability

Data are available from the corresponding author upon reasonable request.
